# Exploring IoT Vulnerabilities in a Comprehensive Remote Cybersecurity Laboratory

**DOI:** 10.3390/s23229279

**Published:** 2023-11-20

**Authors:** Ismael Delgado, Elio Sancristobal, Sergio Martin, Antonio Robles-Gómez

**Affiliations:** 1 Computer Engineering Faculty, Universidad Nacional de Educación a Distancia, 28040 Madrid, Spain; idelgado21@alumno.uned.es (I.D.); arobles@scc.uned.es (A.R.-G.); 2 Industrial Engineering Faculty, Universidad Nacional de Educación a Distancia, 28040 Madrid, Spain; elio@ieec.uned.es

**Keywords:** cybersecurity, Internet of things (IoT), Industry 4.0, remote experimentation, distance laboratories

## Abstract

With the rapid proliferation of Internet of things (IoT) devices across various sectors, ensuring robust cybersecurity practices has become paramount. The complexity and diversity of IoT ecosystems pose unique security challenges that traditional educational approaches often fail to address comprehensively. Current curricula may provide theoretical knowledge but typically lack the practical components necessary for students to engage with real-world cybersecurity scenarios. This gap hinders the development of proficient cybersecurity professionals capable of securing complex IoT infrastructures. To bridge this educational divide, a remote online laboratory was developed, allowing students to gain hands-on experience in identifying and mitigating cybersecurity threats in an IoT context. This virtual environment simulates real IoT ecosystems, enabling students to interact with actual devices and protocols while practicing various security techniques. The laboratory is designed to be accessible, scalable, and versatile, offering a range of modules from basic protocol analysis to advanced threat management. The implementation of this remote laboratory demonstrated significant benefits, equipping students with the necessary skills to confront and resolve IoT security issues effectively. Our results show an improvement in practical cybersecurity abilities among students, highlighting the laboratory’s efficacy in enhancing IoT security education.

## 1. Introduction

In the 21st century, characterized by an increasing dependence on digital technologies, the acquisition of practical cybersecurity skills has become an educational imperative. The rapid proliferation of IoT devices, such as the Arduino, has extended the digital footprint beyond traditional computing devices, thereby elevating the potential for security threats. Yet, the ability to experiment and learn using real-world equipment often remains confined to physical labs due to logistical and financial constraints.

The advent of the “Industry 4.0” paradigm has ushered in novel opportunities and challenges for businesses. To enhance their competitive edge and efficiency, corporations must confront the digital transformation of production and logistics chains (smart manufacturing) and products (smart interconnected objects) [1,2,3]. The profound impact of the fourth industrial revolution spans numerous stakeholders and harbors significant potential for economic growth.

This transformative shift has been made possible due to the concurrent emergence of technologies, such as big data, the Internet of things (IoT), cloud computing, and artificial intelligence. These technologies form the foundation [1,2,3] as they leverage the dimensions of Industry 4.0, enabling interconnectivity and imparting intelligence to new manufacturing systems [4]. Other vital technologies for Industry 4.0, often regarded as pillars alongside the aforementioned ones, include [5] horizontal and vertical software integration, cybersecurity, simulation/digital twins, advanced robotics, additive manufacturing, and augmented reality.

Sectorial studies, as well as national and European policies, acknowledge the crucial role of the fourth industrial revolution in augmenting the competitiveness and modernization of the European manufacturing sector, ultimately propelling economic growth and employability across Europe.

This fourth industrial revolution has also sparked an immense educational challenge [6]. Barely, a decade ago, none of the 4.0 technologies had matured sufficiently to be incorporated into any training program [7]. This calls not only for the education of the current 2.76 million European engineering students but also for the retraining of employees within a sector that encapsulates over 2 million companies and 33 million jobs at the European level.

Current engineering students can master the pillars of Industry 4.0 through conventional on-campus education, but the 33 million employees will likely favor an online method to update their knowledge and skills, owing to professional and familial commitments, as well as time constraints for physically attending on-campus practices [8].

However, ensuring quality online acquisition of practical skills in engineering poses significant challenges [9]. Traditionally, simulators have been commonly utilized in engineering education to support practices [9]. As an evolution, virtual labs (Internet-accessible simulators) and, more recently, remote labs (real hardware accessible through a web interface) emerged, offering time and space flexibility in the learning process and scalability [10].

Distance and online education were the first to reap the benefits of remote laboratories due to the unfeasibility of providing students with practical laboratory practices using real equipment [11]. Later, it also significantly influenced face-to-face education (including primary, secondary, and university) to supplement in-person laboratories [12]. Today, in the wake of the global health crisis caused by COVID-19, these remote laboratories have become a critical factor in the digital transformation of education.

The learning-by-doing approach can deliver complementary competencies to those gained in distance learning courses. The virtual or remote access to laboratories retains the flexibility of the learning model, granting learners unconstrained access in terms of time and space.

Remote laboratories offer a promising solution by providing access to real-world hardware programmed via the Internet. These innovative educational platforms allow students to engage in authentic learning experiences that reflect real-world scenarios, enhancing their ability to tackle contemporary cybersecurity challenges. By combining theory with hands-on practice, remote laboratories can foster an in-depth understanding of cybersecurity, thereby promoting the acquisition of advanced skills and competencies.

This paper underscores the importance of remote laboratories for IoT cybersecurity, utilizing real-world equipment programmed over the Internet, in promoting the acquisition of practical cybersecurity skills, with a particular emphasis on the Arduino-type Internet of things (IoT) devices. It proposes the design of a set of remote practices for a remote IoT cybersecurity lab. The findings from this research will inform the design of effective educational practices in IoT cybersecurity.

This work is organized as follows. A research review of the literature in the topics of the work to find the existing research gaps is presented in Section 2. Section 3 details the methodology employed to achieve our objectives. Section 4 details the proposed remote experiments in IoT and cybersecurity, as well as the principal results obtained. An exhaustive discussion about them is provided in Section 5. Finally, some conclusions and further steps are presented in Section 6.

## 2. State of the Art

The literature analysis reveals that most of the Industry 4.0 educational labs are designed for on-campus experimentation. Among those intended for online experimentation outside laboratory facilities, many were pure simulations or virtual labs.

Limited examples exist of cybersecurity remote labs. Martin and Woodward [12] proposed a “Remote Lab” concept, an accessible solution with initial cost overhead and low maintenance costs. Unlike simulators, a remote lab allows for the observation of nonprogrammed system behavior, bringing an element of unpredictability into the learning process that mimics real-world scenarios.

Willems et al. [13] introduced a “Tele-Lab” platform, which is a hands-on IT security training system within a remote virtual lab ecosystem. A web-based tutoring and training environment was built with virtual machines, offering text, multimedia, and practical exercises. The training process begins with general information, proceeds to a more detailed description of tools and procedures, and concludes with a set of practical exercises. In addition, Willems and Meinel [14] evaluated several practical exercises within an online laboratory, which is based on virtual machine technology. 

Related to the aforementioned labs, Tunc et al. [15] proposed a “CLaaS: Cybersecurity Lab as a Service”, offering a set of virtual cybersecurity experiments accessible in a remote way. The available testbeds include various scenarios, such as a DNS attack, a network packet sniffing experiment, and a DDoS attack, all simulating real-world attack scenarios to enhance the learning efficacy of students.

Salah et al. [16] utilized a cloud computing paradigm (specifically, Amazon AWS) for cybersecurity teaching across two different campuses. The instructor centralized the control over the system. Eight labs were launched to the cloud, covering topics like packet sniffing, network foot-printing and port scanning, vulnerability assessment and penetration testing, backdoor establishment, firewall–EC2 (firewalls hosted within AWS virtual machines), Dionaea honeypot, and OpenSSL.

What is more, Pastor et al. [17] described a system for both cybersecurity and programming environments. The programming module focuses on IoT (edge, fog, and cloud computing), whereas the cybersecurity module focuses only on cybersecurity operations over a virtual shell, not in the IoT programming side of cybersecurity.

Table 1 provides a comparison of the existing remote labs discovered in the literature. It can be observed that none of the evaluated works are focused on IoT cybersecurity. Moreover, upon considering the implementation of these remote labs, it was discovered that none of them are published as open-source to allow any researcher to deploy it. All of them are proprietary solutions, being fully unavailable to the scientific community.

Drawing from this analysis, no other system documented in the literature has been designed to cover an important topic such as online IoT cybersecurity education with remote labs, and even fewer have been publicly released as open-source resources. Such a disruptive system would serve as a formidable catalyst in the digital transition of the industry.

## 3. Materials and Methods

This section describes the methodology followed in this research, as well as the materials (hardware and software) supporting the remote IoT cybersecurity laboratory.

### 3.1. Methodology

The methodology employed in this research consisted of the following steps:

1. Design of the remote laboratory.
2. Identification of threats in the realistic scenario implemented in the remote lab. As there is an intention to evaluate the risk faced due to the malfunctioning of the fan (cooling system), which can be sabotaged by third parties, it is appropriate to identify only those threats that are not caused by natural disasters and industrial sources. These threats are specified in Table 2.

3. Identification of vulnerabilities. Once the main threats are described, the weaknesses or vulnerabilities of our assets can be identified (Table 3).

4. Evaluation of risk. Once the assets, the set of threats, and vulnerabilities are defined, the risk for each asset–threat pair can now be calculated. Since the analysis is qualitative, a risk matrix is used to assess the level of impact on the systems. (Table 4). For our case, a high risk would mean the incorrect functioning of our cooling system. Depending on the type of installation where it is integrated, it can range from a temporary service outage to a catastrophe.

5. Design of practices based on different case studies (one per communication protocol). For each of the defined case studies, a set of direct instructions and guidelines are designed to help future students to acquire specific skills and knowledge in IoT cybersecurity, using the remote IoT cybersecurity lab. Each of these practices or case studies is divided into the following:
  - An introductory section with a brief explanation of how this communication protocol is applied in the remote laboratory.
  - Case study sections, which provide step-by-step instructions for students to execute attacks on the laboratory, exploiting the typical vulnerabilities of each system or communication protocol and developing awareness of possible solutions. During the performance of the proposed activities, unforeseen questions may arise that will serve to reinforce and provide feedback, assisting the student in gaining a deeper understanding of the topic.
  - Each case study concludes with a summary in which inherent vulnerabilities of the communication protocol are identified, as well as those that can be remedied through the use of additional tools or by strengthening the source code of each board’s programs.

### 3.2. Materials—Hardware

Our IoT laboratory consists of three interconnected Arduino boards that serve as the foundation of our experimental setup. These microcontroller boards are renowned for their flexibility and wide-ranging capabilities, making them an ideal platform for IoT scenarios. Each board is equipped with multiple sensors and actuators, facilitating a realistic representation of real-world IoT environments. The interconnection of these boards enables various communication protocols, including a serial port, RS485, I2C, BLE, and Wi-Fi, to be thoroughly examined.

**Figure 1:**
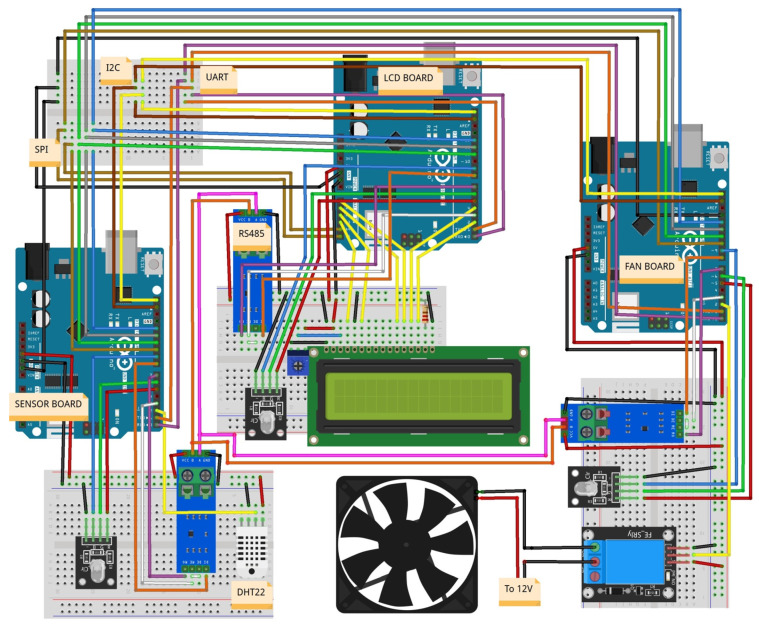
Schematic of the hardware components of the remote laboratory.

The laboratory emulates a basic refrigeration system, utilizing three Arduino Uno WiFi Rev2 boards (see Figure 1):

- Arduino 1 (AR1): This board is connected to a DHT22 temperature and humidity sensor (SAR1).
- Arduino 2 (AR2): This board is attached to an Adafruit 1.8″ TFT Display Shield V2 expansion board and operates as the primary control system. It receives temperature/humidity data and decides whether to activate or deactivate the fan, displaying all relevant information on the TFT screen (SAR2).
- Arduino 3 (AR3): This board has a 12V DC fan connected to it. Given that the Arduino board cannot supply this power directly, an external 12V power source and a transistor are used to deliver power to the fan (SAR3).

The unique design of this laboratory allows for multiple communication channels and protocols for data exchange between the Arduino boards. These include serial port/UART, I2C, SPI, Bluetooth/BLE, Wi-Fi, and RS-485. The chosen Arduino board model does not support this last communication protocol, necessitating the use of a MAX485 expansion module.

Considering the aforementioned design elements, the laboratory’s operational framework can be outlined as follows:

- Arduino 1 (SENSOR):
- Reads sensor data.
- Sends sensor data to AR2.
- Receives reading requests from AR2.
- Arduino 2 (LCD):
- Sends reading requests to AR1.
- Receives sensor data from AR1.
- Sends fan on/off commands to AR3.
- Sends fan status request to AR3.
- Receives fan status from AR3.
- Sends data to the display.
- Arduino 3 (FAN):
- Receives fan on/off commands from AR2.
- Receives fan status request from AR2.
- Sends fan status to AR2.
- Activates or deactivates the fan according to the received command.

As a web server connected to the three Arduino nodes, a Raspberry Pi 3 is used as a low-cost server.

The Arduino boards are on an isolated network, with no possibility of connecting to other institutional networks. Thus, students can work in a secure environment without concerns about compromising institutional cybersecurity.

### 3.3. Materials—Software

**Figure 2:**
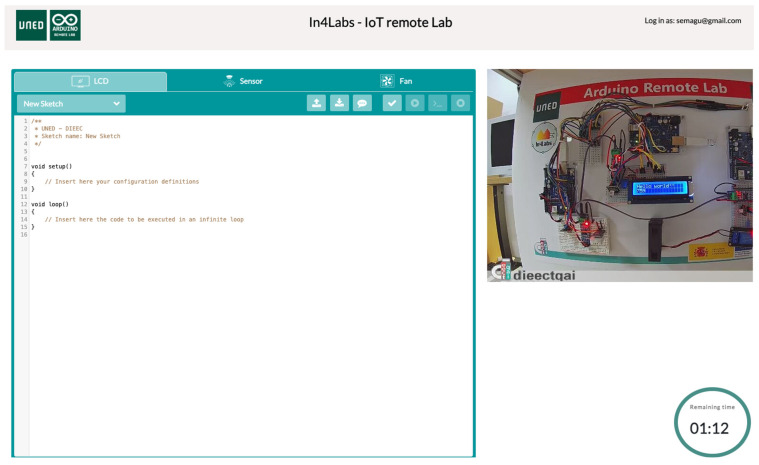
Screenshot of the interface of the remote laboratory.

The laboratory setup is configured to be remotely accessible via a custom-developed software interface (see Figure 2). It is based on the UNED Arduino Remote Labs software (https://github.com/cRejon/in4labs, accessed on 1 November 2023) already used to deploy other remote labs [4]. This allows users to submit the code to be executed in each one of the Arduino boards over the Internet.

The software interface, created for educational purposes, allows users to experiment with and observe the Arduino boards in various scenarios, promoting a deep understanding of potential vulnerabilities and mitigating strategies.

**Figure 3:**
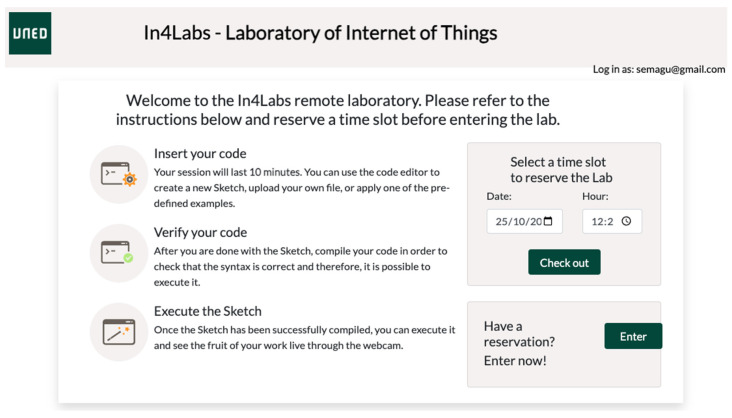
Screenshot of the booking system interface of the remote laboratory.

Students need to book a time slot in the booking system of the remote lab to be able to gain access to the remote laboratory (Figure 3).

When the given time slot (15 min) finishes, the system alerts the student to download his/her code to avoid losing his/her work.

**Figure 4:**
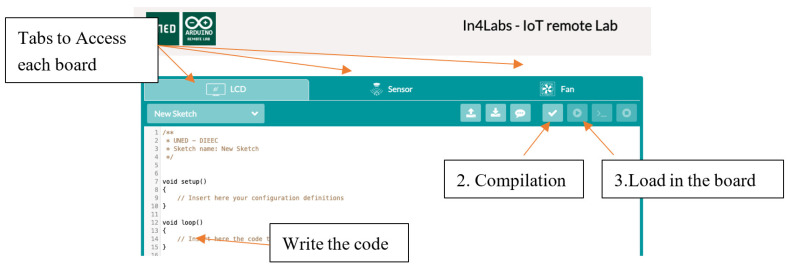
Main buttons of the remote laboratory interface to load code in the boards.

The basic execution flow in the remote lab consists of 3 stages: edit the code, compile it, and execute it in the device (Figure 4).

Regarding the traffic analysis, the Raspberry Pi has tcpdump installed, so it uses the Wi-Fi interface in a promiscuous mode to sniff all the traffic interchanged between the Arduino boards. This information is stored in a file that the student can download and upload to other tools, such as Wireshark, to analyze encrypted traffic.

## 4. Results

This section provides the description of the designed practices for the remote IoT cybersecurity laboratory.

### 4.1. Serial Communication

Serial ports transmit and receive information via bit sequences, requiring two wires and, thus, two connectors: RX (reception) and TX (transmission). When connecting two Arduino boards via the serial port, RX should be connected to TX, and vice versa, in a cross-wired configuration.

Serial ports are physically connected to various Arduino board pins. Using these ports consequently occupies these digital I/Os. In the case of the Arduino board used for this study, pin 0 functions as RX, and pin 1 as TX.

This case study explores an attack scenario involving two Arduino devices communicating through the serial port. While the serial port is commonly used for PC communication, it can also facilitate inter-board communication. This case study is divided into three sections as follows.

#### 4.1.1. Monitorization

This practice consists of one of the Arduino boards monitoring all messages interchanged between the other nodes. In this case, Arduino 3 is in charge of monitoring all the communications interchanged between Arduino 1 and 2. 

The aim is to gain the understanding and awareness of the vulnerabilities inherent in the utilized communication system.

The serial library is used for this case scenario. The following methods are used:

- Begin(speed): to indicate the frequency at which the port communicates.
- Available(): returns the number of bytes available in the port to read and zero in the case there is nothing to read.
- Read(): reads a character from the serial port.
- Write(): writes a character to the serial port.

#### 4.1.2. Supplantation

In this second part, the AR3 board is used to impersonate the sensor data. To achieve this, we only need to connect the RX/TX pair of the serial port and know the message passing between the boards, a task that was already performed in part 1 of this case study. The student can start from the previous code to develop this one.

#### 4.1.3. Cyphering and Denial of Service

As the origin of the attacks is due to the monitoring of the signals that travel through the communication medium, cryptography or encryption can be used to make the information traveling through the medium unreadable and uninterpretable by those who listen to it. It must be clear that the information can still be listened to, and it can also be analyzed, etc., but without an exact understanding of the information that is being received.

In the third part of the case study dedicated to the serial port communication, communications are encrypted using the AES (Advanced Encryption Standard) algorithm. To encrypt and decrypt with the AES in the Arduino, the “AESLib.h” library can be used.

In this case study, the student is asked to check if AR3 can now read the communications interchanged between AR1 and 2. Now, the student understands that thanks to the encryption this is no longer possible.

Nonetheless, even when integrating different security systems into communications, attacks that degrade or block communications can still occur, such as DoS (denial-of service) attacks.

Denial-of-service attacks cause systems, communications, or resources to be inaccessible from other systems or for legitimate users who need access to them for proper operation. In this case, the serial port is the element to attack. If we write continuously to the serial port, the communication between the temperature sensor (AR1) and the master board (AR2) is blocked; thus, a DoS-type attack is carried out.

In this scenario, the student is asked to modify the previous code to carry out a DoS attack on the serial port. This can be achieved by writing in the port without stopping. With this, they can keep the channel occupied and prevent the other boards from communicating with each other.

### 4.2. RS485 Communications

This case study involves monitoring or listening to, interfering with or intercepting, and altering or replacing the communications between two devices, with the aim of causing a malfunction in the system. This case study is divided into three parts as follows.

#### 4.2.1. Monitorization

The communication bus is monitored with the objective of understanding and gaining awareness about the weaknesses in the communication system. 

Communications using the RS485 protocols do not employ any kind of security. These are physical layer protocols responsible for transmitting bits from one station to another. Above this layer, those that provide the protocol with some type of security, authentication, encryption, and addressing should be situated. Having access to the communication bus, we can place a device there to listen to all communications transmitted through it, and since such protocols do not employ security mechanisms, such a device can eventually falsify the sensor data, leading to a catastrophe. This practice asks the student to create a code for monitoring the RS485 communications. 

#### 4.2.2. Supplantation 

Given an invented communication protocol between two devices, the bus is monitored, and false sensor readings are sent, with the aim of causing a failure in the systems.

To control the temperature readings that are taken, so that the receiver indicates its readiness to receive the data, flow control is added to the communication between the devices. One of the boards acts as the master and controls the flow of information; the other board as the slave only sends sensor readings upon the master’s request.

Students are also asked to modify that code to be able to send wrong data supplanting AR1. 

#### 4.2.3. A Man-in-the-Middle Attack

The communication protocol from supplantation is completed, including addressing. The bus is monitored, and an attempt is made to carry out a man-in-the-middle attack.

In this section, addressing is incorporated into the protocol. In this way, two bytes are added to the previous frame format, one to reference the source address and another to specify the destination address. In addition, device discovery instructions are incorporated, so that when the master/slave device is turned on, it sends a frame to identify the components within the bus, using the value 255 as a broadcast address of the bus.

The board that functions as the master is AR2; our protocol has a slave expiration system. In the case a slave does not respond to 10 requests, it is removed from the slave table. Slave devices are added to the table using the discovery frame.

In previous sections, we showed how to monitor the bus and also how to falsify the readings sent by the sensor. In this part, we try to simulate a man-in-the-middle attack.

The goal is not only to listen to the bus communications, but this time, the master is made to believe that the temperature values that arrive are those of the slave they are asked from.

### 4.3. I2C Communications

This case study focuses on carrying out different types of attacks on the work laboratory that communicates using the I2C (inter-integrated circuit) protocol. I2C uses synchronous serial communication, using two cables for this, one for the clock or SCL (system clock) and the other for the data or SDA (system data). On our Arduino boards, the pins are A5 and A4, respectively.

Among the libraries to use for communications with this protocol is “Wire.h” (Arduino-Wire, 2022). Although there are versions of the 7- and 8-bit I2C address protocol, the library used employs 7-bit addresses and uses the eighth bit to establish whether to read or write. When assigning addresses, it should be noted that there are 16 reserved combinations, so the maximum number of nodes to connect is 112 (Internet of things with ESP, 2021).

The implementation of the “Wire.h” library uses a 32-byte buffer, so all communication must be within that limit, as excess bytes are discarded. This case study is divided into four parts as follows.

#### 4.3.1. Slave Discovering

Using functions from the “Wire.h” library, students can see how the addressing of the connected slave devices is discovered.

To carry out this task, the “endTransmission()” method is used. This function ends the data transmission that was initiated with the “beginTransmission(destination_address)” method.

#### 4.3.2. Bus Monitoring

Students learn how to monitor the bus in this case study. The “Wire.h” library for the Arduino’s I2C communications lacks monitoring functions or methods. Therefore, in order to listen to or monitor the bus, the SDA and SCL pair of cables must be connected to digital inputs. Although the rest of the boards do not receive data (since they are addressed), the signal is visible throughout the bus.

#### 4.3.3. Bus Blocking

Once the communication system used in the lab is discovered, an attempt is made to impersonate the sensor by sending an incorrect reading. The AR3 board is used to attempt to send incorrect sensor information. The students are asked to have the AR3 board use the same address as the AR1 board, and with each request from the AR2 board, the AR3 board sends a temperature value of 100.

#### 4.3.4. Cyphering Communications

As students saw in the previous point, once the message passing in the communication system used in the laboratory is discovered, they can impersonate the AR1 board and send incorrect readings. To prevent this, cryptography is used as a method to hide the information sent over the bus, so it cannot be interpreted.

Communications are encrypted using the DES encryption method, and the disadvantages of using this type of encryption is learned.

To use this encryption method in the Arduino, students have to use the “DES.h” library. In the library’s repository, there are examples of how to use DES and 3DES, as the same library encrypts using both methods.

In this case study, the AR1 and AR2 boards communicate by encrypting communications using triple DES.

### 4.4. Bluetooth Low Energy (BLE)

In the current case study, where the boards communicate using the BLE (Bluetooth Low Energy) protocol, the interception of information or message passing between the boards and impersonation attacks are performed.

The BLE protocol is designed for intermittent connection, focusing on establishing connections, transmitting information, and terminating the connection. This strategy significantly reduces energy consumption, enabling a peripheral device with a small button cell battery to last for years. 

This case study is divided into two parts:

- Device scanning. We use methods available in the library to discover sensors and the features they offer.
- Impersonating a peripheral. Once the sensor data is known, we falsify the information available from the sensors to mislead the data received by the master.

The Arduino Uno WIFI Rev2 board, available in our laboratory, integrates a set of chips capable of communicating via Bluetooth and Bluetooth Low Energy, where both the slave and master modes of communication can be implemented. The library used for communication with this protocol is “ARDUINOBLE.h”.

#### 4.4.1. Device Scanning

Passive listening is one of the main security problems in wireless communications, a problem that cannot be solved by the protection methods currently known [18]. Since these boards cannot monitor or sniff on the wireless medium, the methods of the ArduinoBLE library are used to discover information from our boards.

This library integrates a function called “scan” to monitor the environment. Unfortunately, the firmware version of the board and the library version prevent it from working correctly. All the tests performed fail when the method is executed, leaving the laboratory totally blocked. Therefore, the search for devices is performed using the “scanForUuid(UUID)” method.

#### 4.4.2. A Man-in-the-Middle Attack

For the execution of a man-in-the-middle attack with Bluetooth, first, passive listening is performed to discover or intercept the exchange of messages between the devices, since if our intention is to impersonate it, we need to understand the flow of information.

Secondly, students have to make the peripheral, in our case AR1 (the sensor board), believe that it is sending the data to the master. To achieve this, the students keep the connection open or the device connected, making it impossible for another device to connect to it.

Lastly, the students assume the identity of AR1 in AR3, with the same services and characteristics but with incorrect temperature information. The master (AR2) believes that it is connected to the sensor, when in reality it is connected to the attacking board (AR3).

### 4.5. Wi-Fi Communication Case Study

This case study focuses on exploiting vulnerabilities and executing threats against the laboratory while it communicates using Wi-Fi, with the aim of causing system malfunction. This case study is divided into three parts:

- Scanning of Wi-Fi networks, showcasing their characteristics such as name, address, encryption type, etc.
- Attempts to connect to the access point by brute force, using a dictionary of keys.
- A DoS attack by exploiting a vulnerability in the code.

The laboratory is equipped with the Arduino Uno WIFI Rev2 boards, which integrate a chip capable of Wi-Fi communication. In addition, they include an ECC608 cryptographic chip to provide security for the connections. The library used for communications with this protocol is “WiFiNINA.h”). This library allows for the following:

- Creating Wi-Fi instances in server and client modes and sending/receiving UDP packets.
- Connecting to open or encrypted networks.
- Creating open or unencrypted access points, as well as encrypted ones, but only via WPA/WPA2.
- Managing DNS servers.
- Assigning addressing statically or via DHCP.

#### 4.5.1. Scanning of Wi-Fi Networks 

The “WiFiNiNa.h” library can create access points and allows the connection to Wi-Fi networks.

The AR2 board is used to monitor available networks. To achieve this, the “WiFiNINA.h” library is used. The necessary methods to carry out this first part of the case study are as follows:

- canNetworks(): Returns the number of networks found; logically, these are networks that have their SSID published. This method is by default limited to 10 networks. In our case, since there are more networks in the place where the laboratory is located, it is necessary to expand this limit. To achieve this, the value of the constant “WL_NETWORKS_LIST_MAXNUM” defined in the “wl_definitions.h” file located in the library directory must be changed. If no network is found, it will return the value −1.
- SSID(index): Returns the name of the wireless network or SSID of the indicated index.
- encryptionType(index): Returns the type of encryption used by the located network. Possible values are “ENC_TYPE_WEP” for WEP, “ENC_TYPE_TKIP” for WPA, “ENC_TYPE_CCMP” for WPA2, “ENC_TYPE_NONE” for no encryption, “ENC_TYPE_AUTO” for an auto mode, ENC_TYPE_UNKNOWN for an unknown mode.

#### 4.5.2. Attempts to Connect to the Access Point by Brute Force, Using a Dictionary of Keys 

During this part of the case study, an attempt is made to connect to an access point using the attack vector known as brute force. Brute force attack involves systematically checking all possible combinations of a password until the correct one is found. 

This type of attack can be time-consuming and requires a lot of computational resources, but it is one of the simplest methods to gain access to a network or system. It is worth noting that brute force attacks are considered illegal and unethical if they are performed without proper authorization.

In this scenario, the AR1 board is configured as an access point, with SSID “WifiNINA-Access-Point”. The AR2 board is used to perform the attack on the AR1 board, using a dictionary brute force attack vector.

A dictionary brute force attack involves using a pre-compiled list of likely passwords, often based on common words or phrases. The attacker systematically tries each password in the dictionary until they find the correct one. This can be faster than a traditional brute force attack, which tries every possible combination of characters, but it is also less likely to succeed if the password is not a common word or phrase.

To implement this, the AR2 board would need to iterate through each password in the dictionary, attempting to connect to the AR1 board using the WiFiNINA.h library’s WiFi.begin(ssid, pass) function. This function takes as input the SSID of the network to connect to and the password to use for the connection and attempts to establish a connection.

It is important to note that this kind of activity can be legally and ethically problematic. It is critical to only use these techniques in controlled, legal scenarios, such as penetration testing or security research, and only with explicit permission from all parties involved.

The operation of the algorithm is a loop that goes through the dictionary, testing each possible access password. When the connection result is positive, the password is displayed on the screen.

#### 4.5.3. A DoS Attack 

Even with newer, more secure encryption protocols, there are still ways and possibilities for network attacks. Among these are the following:

- Theft of data from old devices: companies sometimes discard devices without first erasing their configurations.
- Spoofing or fake access points: this involves creating access points with names similar to the original to steal information, hijack sessions, etc.
- Injection or repetition of packets: this involves capturing a large number of network packets and distributing them at specific times to alter the operation of the network.
- A deauthentication attack: this involves sending false disassociation packets to an access point that one is connected to, causing it to disconnect temporarily.

DoS: attacks aimed at blocking network devices.

## 5. Discussion

The channel access is usually the most common vulnerability for both wireless and wired networks. This vulnerability involves collision threats, denial-of-service (DoS) attacks, spoofing, etc. To minimize these kinds of attacks, continuous monitoring must be carried out for detecting possible anomalies. Additionally, it is needed to establish user access control from both a logical and physical points of view, in addition to always using encrypted communications. This last aspect is even more relevant when handling wireless communications.

Each one of the presented case studies highlights a set of inherent vulnerabilities. This section discusses them, as well as the information about how to teach to remediate them through the use of additional tools or by strengthening the source code of each board’s programs. Table 5 summarizes the vulnerabilities and mitigation strategies for each communication technology. 

The evaluation and monitoring of each of the activities proposed in the case studies need to be assessed by the instructor. 

### 5.1. Serial Communication Case Study

As demonstrated throughout the case study focused on the serial port, the only method of protecting this communication protocol is to control access to the medium, specifically, the RX/TX wire pair.

The threats encountered in this case study are as follows:

- Traffic analysis.
- Information interception.
- Sequence alteration.
- Denial-of-service attacks.

These threats range from a low risk, such as traffic analysis, to a high risk, like denial-of-service attacks, the latter potentially resulting in the total inoperability of our laboratory. In the case of a cooling system in a nuclear power plant, a failure could lead to an environmental catastrophe.

Given these threats, it is imperative to secure access to the communication wire pair, a concept known as physical security. It is essential to remember that even a low-risk threat like traffic analysis can potentially precede a higher-risk attack. Hence, necessary protection measures include the following:

- Shielding the information flow traveling through the medium. In this case study, communications were protected by encrypting them using the AES algorithm. This measure ensures that even if a malicious user connects to the RX/TX wire pair, they cannot interpret the traffic flow. The threats, in this case, are limited to communication interception and traffic analysis, both considered low risk.
- To protect access to the communication medium, access control mechanisms should be implemented in the facilities. The only way to access the information traveling through the medium is to have physical access to the RX/TX wire pair, as remote access is not feasible.

### 5.2. RS485 Communication Case Study

Given the security issues outlined in this case study, communication between devices using this protocol must be controlled, protecting not just the information flow but also the medium itself. The threats encountered in this case study include the following:

- Traffic analysis.
- Information interception.
- Sequence alteration.
- Spoofing.

Sequence alteration presents a high risk for system malfunction; for instance, if a nuclear power plant’s cooling system fails, it can lead to an environmental catastrophe.

Considering these concerns, both the access to the bus (physical security) and the information flow within it (logical security) must be protected. The following measures should be considered:

- To safeguard the information flow, communications can be encrypted, preventing malicious users connected to the bus from interpreting the traffic flow. Thus, the only threats would be communication interception and traffic analysis, both classified as low risk.
- To secure access to the communication medium, facilities must implement access control mechanisms. As physical access to the bus is the sole means of accessing information in transit, protection against remote intrusion is unnecessary.

### 5.3. I2C Communication Case Study

As observed in the work related to the I2C communication protocol, similar to the serial communication protocol, it does not possess inherent security mechanisms to protect against threats. The threats identified in this case study include the following:

- Traffic analysis.
- Information interception.
- Denial of service.

The only viable countermeasure to these threats remains the protection of the communication medium, in this case, the pair of wires that form the bus. Moreover, by protecting the information in transit through encryption, unauthorized users can be prevented from exploiting it. However, traffic analysis can still be performed, and the bus blockages can occur.

### 5.4. Bluetooth Low Energy (BLE) Case Study

Currently, the ARDUINOBLE library does not have any implemented security system or mechanism to mitigate security risks and potential threats observed during the development of this case study, such as unauthorized device identification and denial-of-service attacks.

The threats encountered can be classified into those that are inevitable, due to the nature of the signal, and others that can be mitigated by enhancing the program controlling the device.

Inevitable threats include those that impact the quality and range of communications, such as electromagnetic interference. Others, like traffic analysis, do not affect signal quality but entail the capture and analysis of network traffic by an attacker. These issues arise because the transmission medium used, i.e., air, cannot be limited or is only restricted by the maximum signal range.

Avoidable threats, such as device impersonation, unauthorized network access, and DoS, can be potentially mitigated by designing and implementing security measures that prevent connections between unauthorized devices. Other specific mitigation strategies might include the following:

- Strengthening device discovery protocols: this means ensuring that devices use secure methods for discovery that prevent unauthorized device tracking or connection attempts.
- Securing communication: implementing secure bonding and pairing procedures and ensuring that data encryption is in place to prevent eavesdropping and impersonation attacks.
- Firmware and library updates: keeping the ArduinoBLE library and the board firmware up to date to fix bugs and security issues that could prevent secure operations.
- Educating on attack methods: understanding the methods attackers use, such as the ones described for impersonating a peripheral, can inform the development of countermeasures.

### 5.5. Wi-Fi Communication Case Study

The current WiFiNINA library enables communication between Wi-Fi networks and, also, allows for the creation of open or closed access points with WPA/WPA2 encryption. It also enables the configuration of devices as clients or servers that will listen to a communication port.

Being a wireless communication protocol, it has the drawback of signal range, which often crosses or transcends the boundaries of an enterprise. This makes the information transmitted via this wireless protocol susceptible to interception by attackers using techniques such as “sniffing” or passive packet capture. Attackers outside the facilities may capture confidential data. Even though data are encrypted, techniques currently exist to decrypt and access the content of each transmission.

The disadvantage of using this type of protocols in operational technology (OT) is that because of the vulnerability of such protocols, additional security measures need to be implemented to make network communications more robust. Some measures, such as always having the latest firmware version, ensure that the discovered security loopholes in the devices are patched.

Other mitigation strategies include continuous network monitoring, which detects suspicious activities, such as a device repeatedly sending data without waiting for a specific period of time. This measure does not focus on resolving the vulnerabilities that the protocol might have but rather attempts to identify the threat and minimize the damage. Also, it is recommended employing strong authentication and encryption measures to protect data integrity and confidentiality over Wi-Fi networks.

## 6. Conclusions and Further Works

This research highlighted the critical vulnerabilities in IoT communication technologies and proposed robust mitigation strategies through the utilization of a remote laboratory framework for cybersecurity education. The investigations into the serial, RS485, I2C, BLE, and Wi-Fi communications revealed various risks ranging from traffic analysis to denial-of-service attacks, all of which pose significant challenges in IoT cybersecurity.

The proposed remote laboratory is a pioneering educational tool that offers a practical and safe environment to explore and mitigate these vulnerabilities. Unlike traditional on-campus labs, the remote laboratory provides a dynamic platform for students to engage in real-world cybersecurity problem solving remotely. The laboratory’s design addresses the most common vulnerability across IoT communication protocols: the channel access. This vulnerability implies the threats of collisions, denial-of-service (DoS) attacks, impersonation, monitoring, etc. 

The only way to mitigate this set of attacks is by the following:

- Monitoring and detecting anomalies.
- Establishing device access control systems.
- Strengthening physical security.
- Encrypting communications.

In terms of scalability and adaptability, the remote laboratory is designed to evolve alongside the cybersecurity landscape. As new threats emerge, the laboratory’s case studies can be updated, and new mitigation strategies can be tested and taught. This ensures that the educational framework remains relevant and effective in teaching the latest security measures. Moreover, the platform’s remote nature allows for rapid updates and modifications, unlike physical labs that may require significant time and financial investment to adapt to new challenges.

This research also underscores the importance of continuous monitoring and updates as part of the cybersecurity protocols. This ongoing process is essential not just for the operational technology but also for educational environments to ensure that learners are always at the cutting edge of security practices. The IoT security challenges identified and addressed in this research through the remote laboratory are pivotal for the development of a resilient and knowledgeable cybersecurity workforce capable of defending against the ever-evolving threats in the IoT ecosystem.

The remote laboratory not only addresses the current challenges in the IoT cybersecurity education but also offers a scalable and adaptable framework for preparing cybersecurity professionals to counter future threats. The laboratory’s ability to simulate and remediate vulnerabilities within a controlled environment makes it a valuable asset in cybersecurity education and contributes to a deeper understanding of the complexities involved in securing IoT technologies.

As future work, authors plan to extend the remote laboratory with new hardware to allow the deployment of new scenarios, such as, for example, with the LoRa technology for longer distances with IoT devices for cybersecurity purposes. Also, other emerging IoT communication technologies such as NB-IoT, Zigbee, Z-Wave, and Sigfox will be considered. Regarding microcontrollers, other hardware platforms will be considered, like ESP32, which has both Wi-Fi and BLE capabilities or even Raspberry Pi Pico for more advanced applications. Regarding new protocols and architectures, MQTT, IPSec, and zero trust architecture will be analyzed. Thus, new practices will have to be developed. For example, to assess the security features and vulnerabilities of protocols like NB-IoT or LoRaWAN, Zigbee, Z-Wave, and Sigfox. These exercises will simulate attacks such as eavesdropping, man-in-the-middle, and replay attacks on these protocols and analyze the outcomes. Also, new scenarios will be developed where students must apply the zero trust principles to IoT networks, ensuring that each device is authenticated and authorized before being granted access to any network resource. These practices would not only enhance students’ understanding of the IoT device security and network setup but also prepare them to think critically about the security implications of integrating different technologies in real-world IoT applications. Finally, game-based methodologies will be applied to improve the learning experience, as it has been previously described in the cybersecurity literature [19,20]. For example, the authors will consider including leaderboards and point systems, simulation of real-world scenarios, story-based learning, and gamified quizzes and assessments.

## Figures and Tables

**Table 1:** Existing cybersecurity remote labs.

Authors	Shell-Based	Cloud	IoT	Full Open-Source
Martin and Woodward [12]	X			
Willems et al. [13]	X			
Willems and Meinel [14]	X			
Tunc et al. [15]	X			
Salah et al. [16]		X		
Pastor et al. [17]	X	X		
Authors			X	X

**Table 2:** Remote IoT lab cybersecurity threats.

Threat	Asset	Description	Dimensions
Sequence alteration	AR1, AR2, AR3	Alteration of the order of received messages with the intention of disrupting the correct functioning of the system	Integrity
Traffic analysis and information interception	Wi-Fi, Bluetooth, RS	The attacker monitors traffic and draws conclusions from the message transit	Confidentiality
Impersonation	AR1, AR2, AR3	The attacker can impersonate a device, making others believe that communications are legitimate	Integrity Confidentiality Availability
Denial of services	AR1, AR2, AR3	An excessive workload causes system failure due to lack of resources	Availability
Software and/or firmware failures	AR1, AR2, AR3	The discovery of such failures can be exploited to access, modify, or control the communication network	Integrity Availability
Sequence alteration	AR1, AR2, AR3	Alteration of the order of received messages with the intention of disrupting the correct functioning of the system	Integrity
Traffic analysis and information interception	Wi-Fi, Bluetooth, RS	The attacker monitors traffic and draws conclusions from the message transit	Confidentiality

Note: Arduino 1 sensor (AR1), Arduino 2 LCD (AR2), and Arduino 3 fan (AR3).

**Table 3:** Remote IoT lab cybersecurity vulnerabilities.

Asset	Vulnerability
AR1, AR2, AR3	Incorrect programming of the Arduino development boards, configuring protocols without any security measures or with weak encryption methods and security issues
AR1, AR2, AR3	Unauthorized access to the devices can imply a reconfiguration of the boards
AR1, AR2, AR3	Lack of patches and updates
Wi-Fi/Bluetooth/RS	Allow unauthorized access to the communication medium
AR1, AR2, AR3	Incorrect programming of the Arduino development boards, configuring protocols without any security measures or with weak encryption methods and security issues

**Table 4:** Remote IoT lab cybersecurity risk by asset–threat.

Asset–Threat	Probability	Impact	Risk
Wi-Fi/Bluetooth/RS–traffic analysis and information interception	medium	medium	medium
AR1/AR2/AR3–supplantation	low	high	medium
AR1/AR2/AR3–sequence alteration	medium	high	high
AR1/AR2/AR3–denial of services	medium	high	high
Software and/or firmware–failure	medium	medium	medium

**Table 5:** Comparison of the vulnerabilities and mitigation strategies of the various communication protocols used in the remote lab.

Communication Technology	Vulnerabilities	Mitigation Strategies	Inherent Security Mechanisms
Serial	Traffic analysis, information interception, sequence alteration, denial-of-service attacks.	Physical security, shielding the information flow, encrypting communications with AES algorithm.	None (control over access to medium).
RS485	Traffic analysis, information interception, sequence alteration, spoofing.	Encrypting communications, physical security, access control mechanisms.	None (protection of information flow and medium itself).
I2C	Traffic analysis, information interception, denial of service.	Protecting the communication medium, encrypting communications.	None (protection of communication medium).
BLE	Unauthorized device identification, denial-of-service attacks, electromagnetic interference, traffic analysis.	Strengthening device discovery protocols, securing communication, firmware and library updates, educating on attack methods.	None (enhancement of program controlling device).
Wi-Fi	Signal interception, susceptibility to sniffing or passive packet capture, vulnerability to attacks from outside facilities.	Continuous network monitoring, strong authentication and encryption measures, firmware updates.	WPA/WPA2 encryption, client/server configuration.

## Data Availability

Data are contained within the article.
